# mTOR plays critical roles in pancreatic cancer stem cells through specific and stemness-related functions

**DOI:** 10.1038/srep03230

**Published:** 2013-11-15

**Authors:** Shyuichiro Matsubara, Qiang Ding, Yumi Miyazaki, Taisaku Kuwahata, Koichiro Tsukasa, Sonshin Takao

**Affiliations:** 1 Cancer and Regenerative Medicine, Frontier Biomedical Science and Swine Research Center, Sakuragaoka, Kagoshima, 890-8520, Japan; 2 Department of Digestive Surgery, Kagoshima University Graduate School of Medical and Dental Sciences 8-35-1, Sakuragaoka, Kagoshima, 890-8520, Japan

## Abstract

Pancreatic cancer is characterized by near-universal mutations in KRAS. The mammalian target of rapamycin (mTOR), which functions downstream of RAS, has divergent effects on stem cells. In the present study, we investigated the significance of the mTOR pathway in maintaining the properties of pancreatic cancer stem cells. The mTOR inhibitor, rapamycin, reduced the viability of CD133^+^ pancreatic cancer cells and sphere formation which is an index of self-renewal of stem-like cells, indicating that the mTOR pathway functions to maintain cancer stem-like cells. Further, rapamycin had different effects on CD133^+^ cells compared to cyclopamine which is an inhibitor of the Hedgehog pathway. Thus, the mTOR pathway has a distinct role although both pathways maintain pancreatic cancer stem cells. Therefore, mTOR might be a promising target to eliminate pancreatic cancer stem cells.

Pancreatic cancer is a lethal disease because current chemotherapies such as gemcitabine provide negligible survival benefits for this cancer. Accumulating evidence suggests that a small population of cancer stem cells (CSCs) in malignant tumors is responsible for sustaining tumor formation, growth, metastasis and recurrence^1^,^2^,^3^. Conventional chemotherapy and radiotherapy only affect rapidly growing cancer cells that constitute the bulk of a tumor. Such therapies can reduce tumor mass, but they cannot prevent recurrence, indicating their failure at eliminating CSCs. It is often reported that treatment with radiation and anti-cancer drugs results in the enrichment of CSCs^4^,^5^,^6^,^7^. Therefore, new strategies targeting cancer stem cells are essential to improve pancreatic cancer therapies. The signaling pathways that function to maintain CSC properties have become the focus of the search for novel therapeutic targets. The inhibition of these pathways might be an effective approach to eliminate CSCs.

Pancreatic cancer is characterized by near-universal mutations in KRAS and frequent deregulation of crucial embryonic signaling pathways, such as the Hedgehog and Wnt-β-catenin pathways. Aberrant activation of these pathways is involved in the progression of pancreatic cancer^8^. The phosphatidylinositol 3-kinase (PI3K)/Akt/mammalian target of rapamycin (mTOR) pathway is activated downstream of RAS signaling and likely represents a major mediator of RAS-driven oncogenesis^9^,^10^. In human pancreatic cancer, the PI3K/Akt/mTOR pathway is deregulated in the majority of tumors^11^,^12^,^13^, and the activation of this pathway correlates significantly with a poor prognosis^14^. Based on these findings, these signaling pathways are potential candidates for targeted therapies.

In the present study, we focused on the mTOR pathway based on the results of our screening for potential agents effective against pancreatic cancer stem-like cells (see Results section). mTOR is the target of a complex signal transduction pathway known as the PI3K/Akt/mTOR cascade. This pathway is highly branched and activates mTOR, a serine/threonine protein kinase, among other downstream effectors. The mTOR kinase assembles into at least two distinct complexes called mTOR complex 1 (mTORC1) and mTOR complex 2 (mTORC2), each of which has unique substrates. mTORC1 is composed of mTOR, regulatory-associated protein of mTOR (Raptor), and mammalian LST8/G-protein β-subunit like protein (mLST8/GβL). This complex is directly inhibited by rapamycin. mTORC2 is composed of mTOR, rapamycin-insensitive companion of mTOR (Rictor), mLST8/GβL, and mammalian stress-activated protein kinase interacting protein 1 (mSIN1). Rapamycin does not appear to be a general inhibitor of mTORC2; however, in a subset of human cancer cells, rapamycin does inhibit mTORC2 by preventing its assembly. The determinants of this phenomenon are unknown^15^,^16^.

The PI3K/Akt/mTOR pathway has diverse effects on stem cells. This pathway is important for the proliferation, survival and maintenance of pluripotency in ES cells^17^,^18^,^19^. Studies in mTOR knockout mice have shown that mTOR is essential for early blastocyst formation and ES cell proliferation^20^,^21^. Rapamycin augments the differentiation of ES cells^22^. The activation of this signaling pathway by the deletion of phosphatase and tensin homolog (Pten), which antagonizes the function of PI3K, increases cell cycle entry and self-renewal in neural stem cells^23^,^24^,^25^. Blocking both mTOR and PI3K promotes the differentiation of glioblastoma stem-like cells^26^. These findings are in agreement with the hypothesis that the mTOR pathway maintains the stem cell-like properties of pancreatic CSCs.

Here, we report that inhibiting the mTOR pathway suppressed the growth of CD133-expressing (CD133^+^) pancreatic cancer cells and reduced pancreatic cancer cell sphere formation under stem cell culture conditions and colony formation in soft agar. These findings suggest that the mTOR pathway plays an important role in the self-renewal of pancreatic CSCs. We also discuss the specific function of the mTOR pathway by comparing the effects of mTOR inhibition with the effects of Hedgehog signaling inhibition.

## Results

### The mTOR inhibitor rapamycin does not affect the content of CD133^+^ cells but significantly reduces the overall viability of pancreatic cancer cells, indicating the elimination of CD133^+^ cells

**Figure 1:**
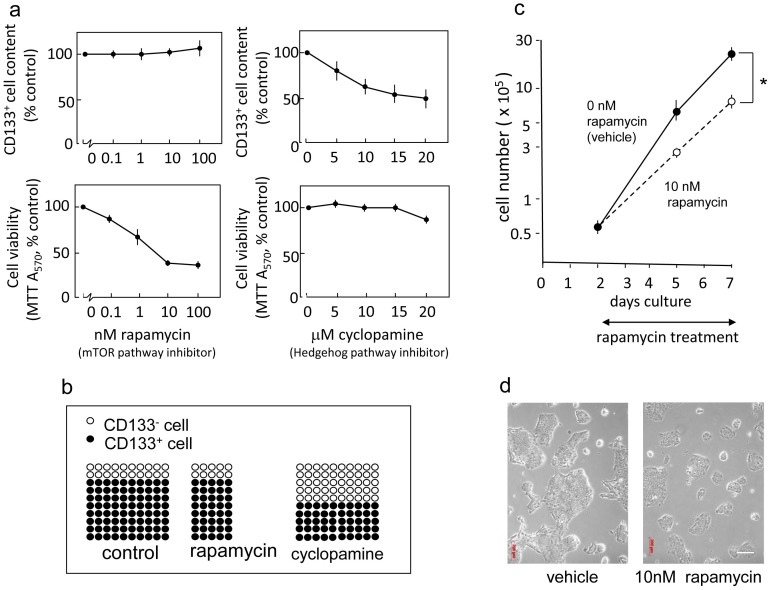
The mTOR inhibitor rapamycin does not affect the frequency of CD133^+^ cells but significantly reduces the viability of pancreatic cancer cells. (a) Capan-1M9 cells were treated with the inhibitors at the indicated concentrations for three days. The effects of the mTOR inhibitor rapamycin and the Hedgehog pathway inhibitor cyclopamine on CD133^+^ cell frequency and cell viability were determined by flow cytometry and the MTT assay, respectively. The results are presented as percentages of control values in untreated cells, showing the mean and s.d. of four replicates obtained in one representative experiment out of three. (b) Schematic representation of the effects of rapamycin and cyclopamine on cell viability of CD133^+^ and CD133^−^ cells. The vertical axis indicates the CD133^+^ cell frequency, and the horizontal axis indicates the total cell viability. The closed circles represent the CD133^+^ cells, and the open circles represent the CD133^−^ cells. (c) The mTOR inhibitor rapamycin suppresses the growth of Capan-1M9 pancreatic cancer cells. The cells were seeded at an initial density of 10^5^ cells per 35-mm dish. Rapamycin was administered two days after plating, and the cell numbers were then determined over a period of five days using trypsin/EDTA treatment. The vertical axis is shown on a logarithmic scale. All data are the mean ± s.d. *P < 0.01. (d) Morphology of Capan-1M9 cells untreated (*left*) and treated (*right*) with 10 nM rapamycin for three days. Scale bar, 200 μm.

We recently established a highly migratory and invasive subclone called Capan-1M9 from the human pancreatic cancer cell line Capan-1^27^. This subclone displays elevated expression of CD133, and approximately 80–90% of the cells express CD133 (Supplementary Figure S1 and Ref. 27). Because CD133^+^ Capan-1 cells were identified as a population of cancer stem-like cells (Supplementary Figure S2 and Ref. 28), we sought to use this subclone to screen for potential agents effective against CD133^+^ pancreatic cancer cells. We treated Capan-1M9 cells with inhibitors of signaling pathways that are important for embryonic development or the regulation of stem cells, and then we analyzed the percentage of CD133^+^ cells by flow cytometry and cell viability by MTT assay. We found that rapamycin did not affect the percentage of CD133^+^ cells but significantly reduced total cell viability (Figure 1a). This effect was clearly different from the effects of Hedgehog inhibition: cyclopamine, an inhibitor of the Hedgehog pathway, significantly reduced the percentage of CD133^+^ cells, but it did not reduce the viability of Capan-1M9 cells (Figure 1a). These results suggest that the two pathways act through different mechanisms to maintain CD133^+^ cells.

In addition, the effect of rapamycin is different from that of gemcitabine, the standard treatment for pancreatic cancer. We previously reported that treatment of Capan-1 cells with gemcitabine increased the content of CD133^+^ cells^28^. A marked enrichment of CD133^+^ cells following gemcitabine treatment was also demonstrated using other cell lines derived from pancreatic cancers^3^,^29^. These results were explained by the notion that CD133^+^ cancer stem-like cells are resistant to anticancer drugs such as gemcitabine. Gemcitabine eliminates CD133^−^ cells but does not reduce the viability of CD133^+^ cells, and as a result, an increased percentage of CD133^+^ cells was observed after treatment (see Supplementary Figure S2b, c). In contrast, rapamycin effectively reduced the viability of Capan-1M9 cells, which contain 80–90% CD133^+^ cells. Therefore, our results suggest that rapamycin can eliminate both CD133^+^ cancer stem-like cells and CD133^−^ cancer cells, resulting in no net increase in the frequency of CD133^+^ cells (Figure 1b).

The growth inhibitory effect of rapamycin on Capan-1M9 cells was confirmed by growth curve analysis based on direct cell counts as well as MTT assays. The cells continued to proliferate in the presence of 10 nM rapamycin, but the growth rate was reduced by half (Figure 1c). The cell morphology was not significantly altered (Figure 1d). In addition, inhibitors of Wnt or Notch did not significantly affect either the percentage of CD133^+^ cells or the viability of Capan-1M9 cells (Supplementary Figure S3).

### Reduced CD133 expression does not affect cell viability following rapamycin treatment

**Figure 2:**
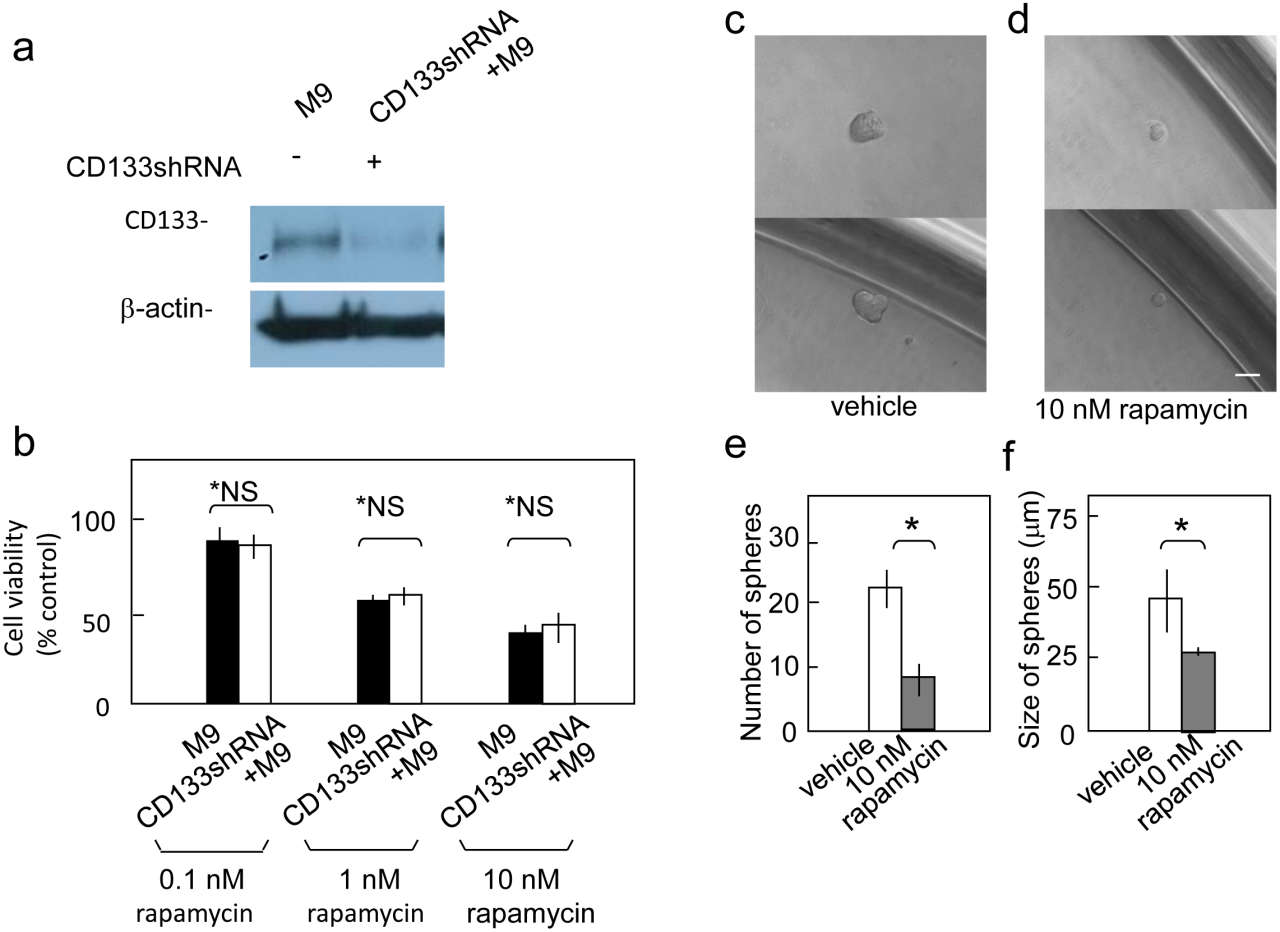
Reduced CD133 expression does not affect cell viability after rapamycin treatment. (a) Immunoblot detection of CD133 polypeptide in Capan-1M9 cells expressing the CD133 shRNA (shCD133M9). The full-length blots are shown in the supplemental Figure S4. (b) Capan-1M9 cells expressing (open bars) and not expressing (closed bars) the CD133 shRNA were treated with the indicated concentrations of rapamycin for three days. Cell viability was determined, as shown in Figure 1(a). All data are the mean ± s.d. *NS, not significant by a two-tailed Student's *t*-test. Rapamycin inhibits sphere formation in Capan-1M9 cells. (c) and (d) Representative photographs of spheres cultured in stem cell medium in the absence (c) or presence (d) of 10 nM rapamycin. Scale bar: 50 μm. (e) Single-cell suspensions (0.7 cells/100 μl) were plated in wells and cultured for eight days. The number of spheres formed per 96-well plate was determined in triplicate plates. All data are the mean ± s.d. *P < 0.01. (f) The cells were cultured as in (e), and the sizes of the spheres were determined. All data are the mean ± s.d. *P < 0.05.

Because rapamycin reduced the viability of both CD133^+^ and CD133^−^ pancreatic cancer cells, we expected that decreasing CD133 expression via shRNA would not affect the efficacy of rapamycin treatment. shCD133M9 cells are Capan-1M9 cells transfected with a CD133shRNA expression construct to knock down endogenous CD133^27^ (Figure 2a). The viability of the shCD133M9 cells was similar to that of the control Capan-1M9 cells after rapamycin treatment, indicating that the sensitivity to rapamycin is not affected by CD133 expression itself (Figure 2b). This result is consistent with the above hypothesis. However, the possibility remains that CD133 expression is a marker of CSCs, even though CD133 may not have any functional roles in maintaining CSC properties.

The screening revealed that the mTOR inhibitor rapamycin can effectively reduce the growth and/or viability of CD133^+^ pancreatic cancer cells. The effect of rapamycin is unique in comparison with the anticancer drug gemcitabine or the Hedgehog inhibitor cyclopamine because it does not alter the frequency of CD133^+^ cells among Capan-1M9 cells.

### Rapamycin inhibits pancreatic cancer cell sphere formation

The above finding is attractive as a potential basis for a CSC-targeting therapy. However, in the above experiments, CSC-like cells were identified based on CD133 expression rather than directly by cellular function. Hence, it was important to examine the connection between the mTOR pathway and the functional properties of CSCs. To assess this connection, we analyzed the effect of rapamycin treatment on sphere formation. Spheroid formation is an index of self-renewal capacity, which is a major property of stem cells^30^. Cancer stem-like cells have been cultured under bFGF(+)EGF(+) serum-free conditions to investigate their sphere-forming ability^31^,^32^,^33^,^34^. In the present study, to more accurately examine clonogenicity under stem cell growth conditions, we cultured pancreatic cancer cells in 96-well plates using the limiting dilution method. Under stem cell growth conditions, Capan-1M9 cells formed spheres that reached 45 μm in diameter after eight days (Figure 2c). The administration of 10 nM rapamycin reduced sphere formation in both number and size (Figure 2d, e and f). Thus, the mTOR pathway is functionally linked to the self-renewal of cancer stem-like cells.

**Figure 3:**
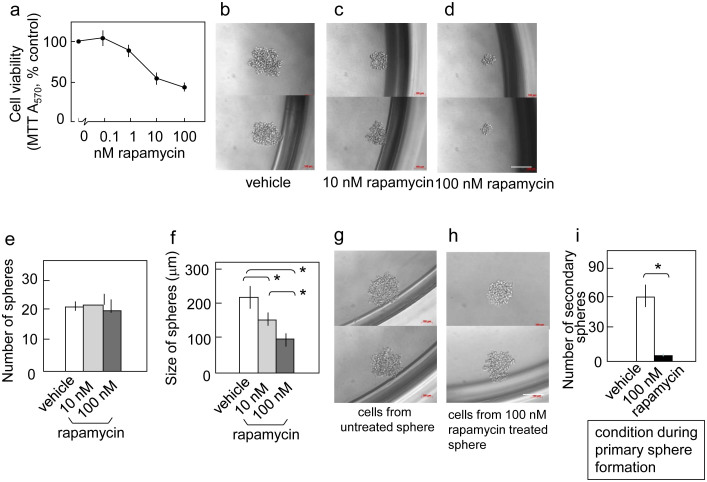
mTOR inhibition reduces the self-renewal of cancer stem-like cells in PANC-1 cells. (a) Rapamycin reduced the viability of PANC-1 cells in the two-dimensional culture. PANC-1 cells were treated with the indicated concentrations of rapamycin for three days. Cell viability was determined as indicated in Figure 1(a). Representative photographs of spheres (primary spheres) cultured in the stem cell medium in the absence (b) or presence of 10 nM (c) and 100 nM (d) rapamycin. Scale bar: 200 μm. (e) Rapamycin does not affect the number of primary spheres. The cells were cultured and assayed, as described in Figure 2(e). (f) Rapamycin reduces the size of primary spheres. The cells were cultured as in (e), and the results are indicated in Figure 3(f). (g) and (h) Representative photographs of secondary spheres. Single cells from primary spheres that were untreated (g) or treated with 100 nM rapamycin (h) were plated in 96 wells and then cultured without rapamycin for eight days. Scale bar: 200 μm. (i) Rapamycin reduces the self-renewal capacity of stem-like cells, as reflected by the formation of secondary spheres. Single cells from primary spheres were seeded in 96-well plates and cultured without rapamycin for eight days. The indicated results are the mean values and s.d. of the number of secondary spheres formed per 96-well plate. All data are the mean ± s.d. *P < 0.01.

To further ascertain the role of the mTOR pathway in the self-renewal of pancreatic cancer stem-like cells, we analyzed another pancreatic cancer cell line, PANC-1. PANC-1 lacks CD133 expression but has been shown to form tumors in immunodeficient mice (data not shown). Rapamycin reduced the viability of PANC-1 cells in two-dimensional cultures, showing a similar but slightly weaker effect than that observed in Capan-1M9 cells (Figure 3a). Under stem cell culture conditions, PANC-1 cells formed spheres of 200 μm in diameter after eight days (primary sphere, Figure 3b). The administration of 10–100 nM rapamycin reduced sphere size in a concentration-dependent manner (Figure 3c, d and f), whereas it did not reduce the number of spheres (Figure 3e).

To further analyze the function of mTOR in the self-renewal of stem-like cells in the PANC-1 cell line, we analyzed secondary sphere formation. PANC-1 cells were grown under stem cell culture conditions with or without 100 nM rapamycin. Then, single cells were prepared from these primary spheres, and their sphere-forming capacities were compared. In this experiment, primary spheres formed in a 96-well plate were combined and processed together, and the resulting single cells were recovered in the same volume. Equal volumes of cell suspensions from each treatment were then analyzed for sphere formation under rapamycin-free conditions. The rapamycin treatment reduced the number of secondary spheres (Figure 3i), indicating that inhibiting the mTOR pathway during primary sphere formation reduced the number of stem-like cells that had the ability to form secondary spheres. As shown in Figures 3g and 3h, the rapamycin treatment did not affect the size or shape of the secondary spheres, suggesting that the stem-like cells in the rapamycin-treated spheres have similar properties to the cells in the untreated spheres. These data show that the mTOR pathway is functionally linked to the self-renewal of cancer stem-like cells not only in Capan-1M9 cells but also in PANC-1 cells.

**Figure 4:**
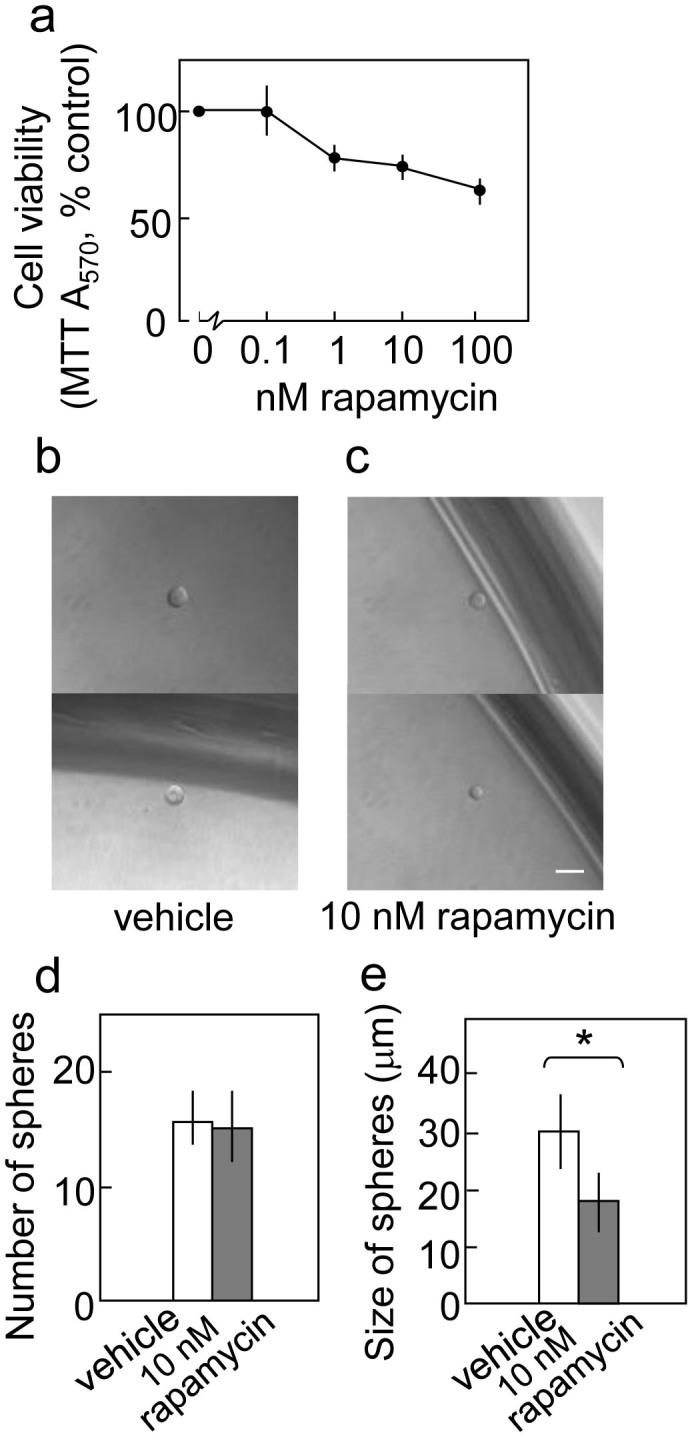
mTOR inhibition reduces sphere growth in the recently established pancreatic cancer cell line PCK-2. (a) Rapamycin reduced the viability of PCK-2 cells in the two-dimensional culture. Cell viability was determined, as indicated in Figure 1(a). Representative photographs of the spheres cultured in the stem cell medium in the absence (b) or presence (c) of 10 nM rapamycin. Scale bar: 50 μm. (d) Rapamycin does not affect the number of spheres. The cells were cultured, as shown in Figure 2(e). The indicated results are the mean values and s.d. of the number of spheres formed per 288-wells (three 96-well plates) in triplicate experiments. (e) The cells were cultured as in (d), and the sizes of the spheres were determined. All data are the mean ± s.d. *P < 0.05.

PCK-2 is a novel cell line derived from the ascites of a pancreatic adenocarcinoma patient. Cell lines established freshly from tumors may reflect the behavior of cancer cells in patients more accurately than the cell lines which maintained in vitro for long time. PCK-2 cells that were injected into NOD/SCID mice generated tumors that histologically resembled the original tumor (data not shown), suggesting that PCK-2 contains a subpopulation of CSCs. Rapamycin (100 nM) reduced the viability of PCK-2 cells in two-dimensional cultures by 37% (Figure 4a). Under stem cell culture conditions, PCK-2 cells formed spheres of 30 μm in diameter after eight days (Figure 4b). The administration of 10 nM rapamycin reduced the sphere size (Figure 4c and e) but not the number of spheres (Figure 4d), suggesting that the effect of rapamycin treatment on PKC-2 cells is similar to that on PANC-1 cells.

Taken together, these findings demonstrate that rapamycin inhibits sphere formation in three pancreatic cancer cell lines, confirming that the mTOR pathway is important for stem-like cell functions.

### Rapamycin inhibits anchorage-independent growth of pancreatic cancer cells

**Figure 5:**
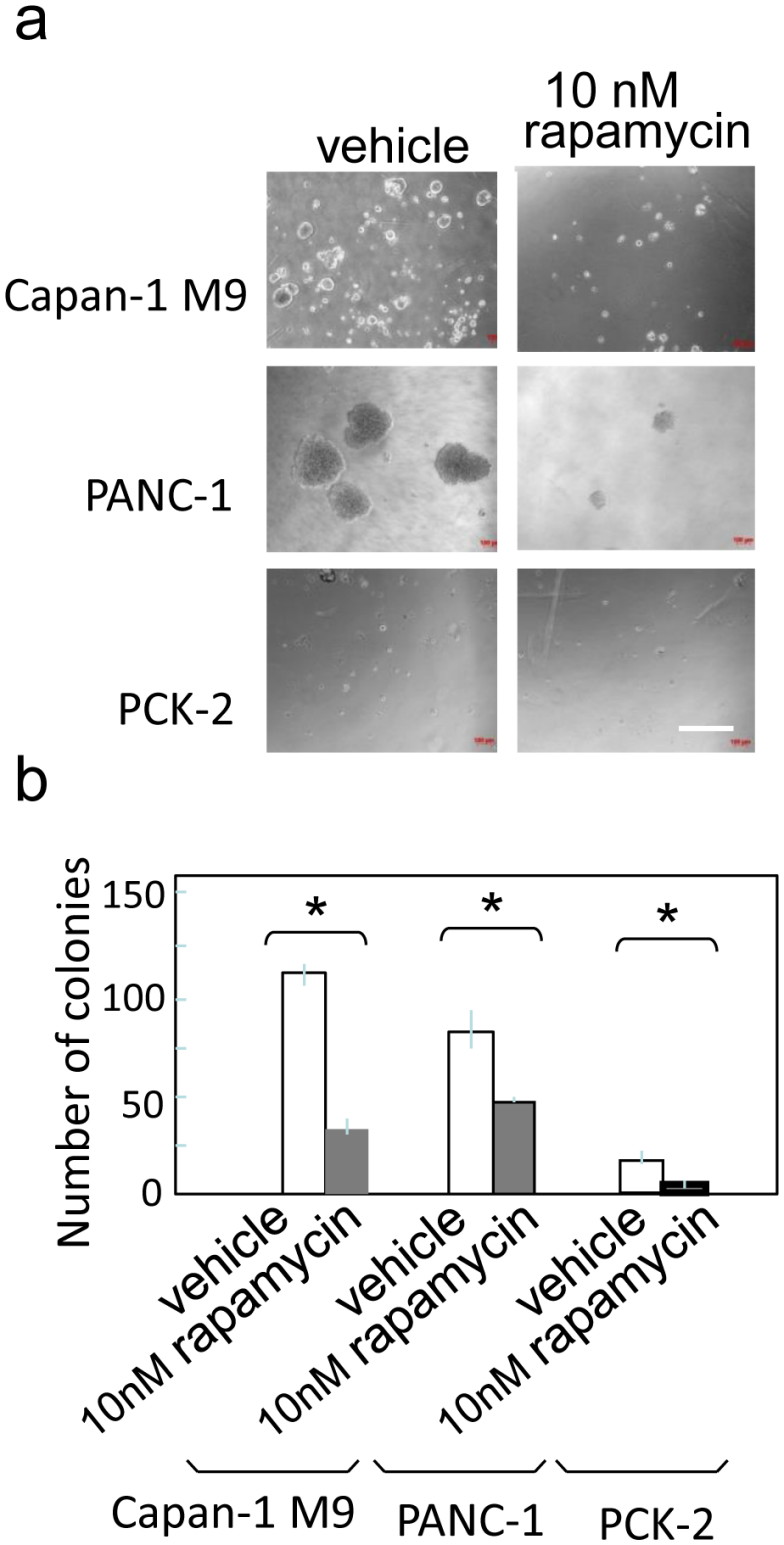
Rapamycin inhibits anchorage-independent colony formation in pancreatic cancer cells. (a) Representative photographs of colonies in soft-agar in the absence (left) or presence (right) of 10 nM rapamycin. Scale bar: 400 μm. (b) Single cells in soft agar were seeded in 24-well plates and cultured for two weeks. The number of colonies formed per well is indicated as the mean ± s.d. from three wells in one representative experiment out of three. *P < 0.05.

Colony formation in soft agar is an index of malignancy and has recently been linked to CSC properties^30^,^32^. We analyzed the effects of rapamycin on colony formation in Capan-1M9, PANC-1, and PCK-2 cells. Inhibition of the mTOR pathway by 10 nM rapamycin reduced the number of colonies formed in soft agar by the three cell lines (Figure 5a and b). This finding further supports the functional relationship between the mTOR pathway and the maintenance of cancer stem-like cell properties.

### Phosphorylation and activation status of mTOR effectors after rapamycin treatment

**Figure 6:**
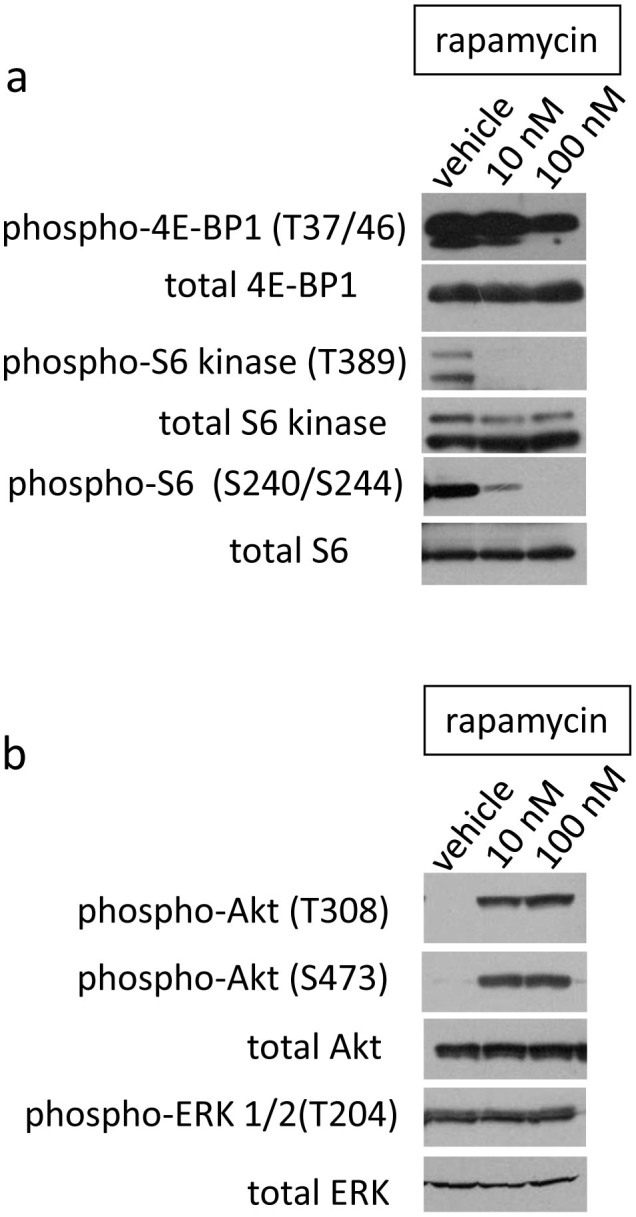
Phosphorylation of mTOR effectors after rapamycin treatment. (a) Phosphorylation and activation of mTOR downstream effectors in Capan-1M9 cells. (b) Phosphorylation and activation of Akt and ERK in Capan-1M9 cells. Capan-1M9 cells were cultured and treated with the indicated concentrations of rapamycin. The cell lysates were immunoblotted to detect the indicated proteins. The full-length blots are shown in the supplemental Figure S5.

To confirm the effects of rapamycin on mTOR signaling, we quantitatively evaluated the phosphorylation and/or the activation status of mTOR effectors by immunoblotting (Figure 6). The phosphorylation of the downstream effector of mTOR 4E-BP1 (at sites T37/46) was reduced by rapamycin treatment. The phosphorylation of the direct mTOR target p70/p85 S6 Kinase (S6K, at site T389) and its substrate S6 ribosomal protein (at sites S240/244) was strongly inhibited by rapamycin (Figure 6a). These results indicate that rapamycin negatively regulates the phosphorylation of effectors downstream of mTOR (mTORC1) and inhibits the mTOR signaling pathway.

The phosphorylation of Akt was also examined because of a known feedback loop affecting the PI3k/Akt/mTOR pathway after mTORC1 is inactivated upon inhibition of Grb10 and/or activation of IRS^35^,^36^,^37^. The phosphorylation of Akt was increased at both T308 and S473 following rapamycin treatment (Figure 6b). Akt requires phosphorylation at these two sites for full activation^16^. Because S473 is phosphorylated by mTORC2^38^, this finding might indicate that mTORC2 activity was increased in the rapamycin-treated cells. The phosphorylation status of ERK1/2 was not affected by rapamycin, although crosstalk between the PI3k/Akt/mTOR pathway and the MEK/ERK pathway has been reported in glioblastoma stem-like cells^39^.

### Not only mTORC1 but also mTORC2 are involved in the mTOR function to maintain stem-like property of pancreatic cancer cells

To analyze the significance of mTORC2 in the maintenance of pancreatic CSC-like properties, we examined the effect of ATP-competitive and mTORC1/mTORC2 dual inhibitor KU-0063794 and the results were compared to rapamycin and a rapamycin derivative everolimus. Rapamycin and its derivatives directly inhibit mTORC1 but not mTORC2, although in some cases but not generally they inhibit mTORC2 by preventing its assembly (16). The effect of everolimus on viability of Capan-1M9 cells was very similar when the difference of IC50 of kinase activity is considered (Supplementary Figure S6a). IC50 of rapamycin and everolimus are 0.1 nM and 1.6–2.4 nM respectively. In contrast KU-0063794 was less effective in lower concentration at 100 nM than rapamycin (IC50 of KU-0063794 is 10 nM), and at high concentration 10000 nM (10 μM) showed 90% inhibition (Supplementary Figure S6b), showing a contrast to the inhibition by rapamycin which reaches plateau around 60% at 10 nM. This pattern of inhibition caused by KU-0063794 is different from that of rapamycin. In the sphere formation assay similar difference was observed in KU-0063794 and rapamycin treated cells (Supplementary Figure S6c). These results indicate that not only mTORC1 but also mTORC2 are involved in the mTOR functions to maintain stem-like properties of pancreatic CSCs.

### Effect of rapamycin on xenograft tumors

**Figure 7:**
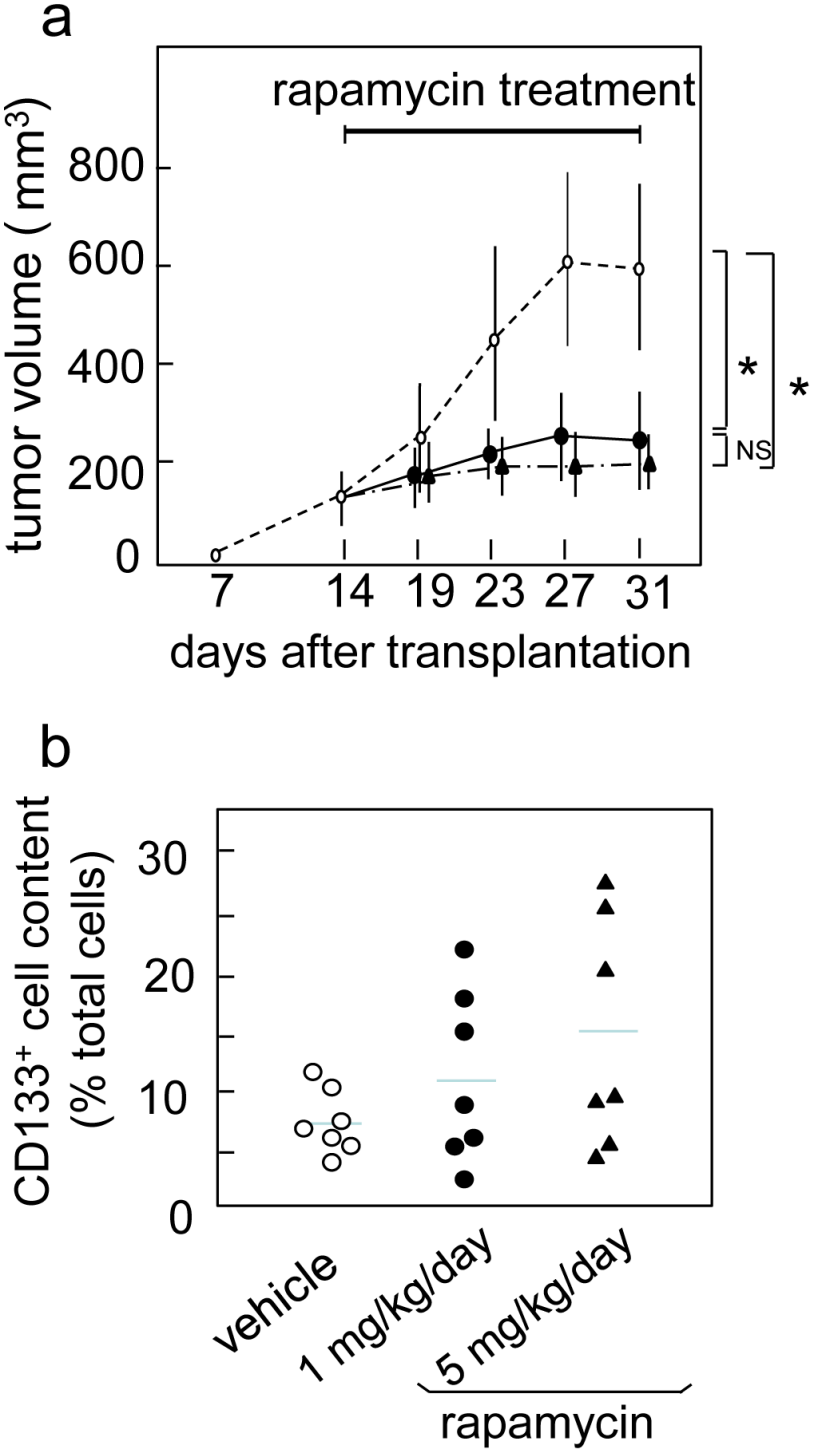
mTOR inhibitor rapamycin impairs *in vivo* growth of xenografted pancreatic carcinoma in nude mice. (a) Tumor growth of Capan-1M9 xenografts which were treated with vehicle (open circle), 1 mg/kg/day (closed circle), or 5 mg/kg/day (closed triangle) rapamycin. Tumor volume was shown as the mean and SD from 5 mice (10 tumors) from each group. Rapamycin treatment showed a significant effect. * *P* < 0.01. (b) The variance in the percentage of CD133^+^ cells after the flow cytometric analysis of xenografts.

To evaluate the effect of mTOR inhibition *in vivo* and compare it with the results in vitro, we administrated rapamycin to nude mice bearing subcutaneous xenograft tumors derived from Capan-1M9 cells. Rapamycin treatment suppressed tumor growth at 1 mg/kg/day (Figure 7a and Supplementary Figure S7a) and led to significant difference in tumor growth curve compared to the control (p < 0.01, after 17 days treatment). The increase of daily dosage to 5 mg did not caused the significant change (p > 0.05). The flow cytometric analysis of cells prepared from xenografts showed the tendency that the percentage of CD133^+^ cells increased after rapamycin treatment (Figure 7b and Supplementary Figure S7c), although the difference was not statistically significant (p = 0.118 between control and 1 mg/kg/day, p = 0.085 between control and 5 mg/kg/day). The increased variance in the percentage of CD133^+^ cells were observed in rapamycin treated xenografts (Figure 7b). The body weight did not change so significantly (Supplementary Figure S7b).

## Discussion

Few reports have described the function of mTOR in pancreatic CSCs. Mueller et al.^29^ reported that combined treatment using Hedgehog inhibitors (cyclopamine/CUR199691) and the mTOR inhibitor rapamycin in addition to gemcitabine effectively eliminated tumorigenic CSCs in human pancreatic cancer. In this report, the authors demonstrated that the inhibition of both Hedgehog and mTOR reduced the frequency of CD133^+^ cells in the L3.6pl cell line after a single treatment, and they discussed the possibility that successful elimination of CSCs may require the inhibition of multiple stemness pathways due to the redundancy and/or non-exclusiveness of the pathways.

Rapamycin treatments reduced the CD133^+^ cell content of L3.6pl cells in their experiments but did not reduce the content of Capan-1M9 cells in our results. The specific reason for this discrepancy in response has not been identified; however, one possible explanation is that the two cell lines have different background characteristics. For example, 2.73% of L3.6pl cells are CD133^+^, whereas approximately 80–90% of Capan-1M9 cells are CD133^+^. In addition, the L3.6pl cells were selected in vivo as metastatic variants from COLO 357 cells^40^, whereas the Capan-1M9 cells were selected in vitro as highly migratory cells from Capan-1 cells^27^. In the parental cell lines, genetic alterations of p16^INK4a^ and p53 are absent in COLO 357 cells, whereas homozygous deletion of p16^INK4a^ and mutation (159 Val) of p53 have been reported in Capan-1 cells^41^,^42^,^43^,^44^. In addition, homozygous deletion of DPC-4 (SMAD4) has been detected in COLO 357 cells but not in Capan-1 cells. Instead, a point mutation (343stop) was detected in this gene in Capan-1 cells^45^.

These differences in genetic background may affect cellular response to rapamycin and could be a reason for the discrepancy between our results and those of Mueller et al. Moreover, it should be noted that the p16^INK4a^/p14^ARF^ (p19^Arf^ in mouse), p53, and DPC-4 (SMAD4) genes are frequently mutated in pancreatic cancer^46^. Inactivation of the tumor suppressor genes p16^INK4a^ and p53 is correlated with the pathogenesis of ductal adenocarcinoma in 38–82% and 33–76% of primary cancers, respectively^47^,^48^,^49^. Therefore, our findings in Capan-1M9 cells, which contain the p16^INK4a^ deletion and the p53 mutation, are important to the understanding of the pathogenesis of pancreatic cancer.

Generally, changes in CD133^+^ cell content are easier to detect in cell populations with high contents of CD133^+^ cells. However, we could not detect any reduction in CD133^+^ cell content in Capan-1M9 cells after rapamycin treatment. Therefore, our results indicate that rapamycin treatment does not always reduce the percent of CD133^+^ cells. Importantly, a combination analysis of CD133^+^ cell content and total cell viability indicated that the viability of CD133^+^ cells was decreased by rapamycin treatment (Figure 1b), which suggests that a reduction in CD133^+^ cell frequency was not necessary for the elimination of CD133^+^ cells. To explain this result, we propose that rapamycin reduced the viability of not only CD133^+^ cells but also CD133^−^ cells. We confirmed that CD133 expression itself did not affect cell viability after rapamycin treatment.

In the present study, rapamycin reduced the viability of CD133^+^ cells in the Capan-1M9 cell line and inhibited sphere formation in three different pancreatic cancer cell lines. The latter is an index of the self-renewal of stem-like cells. The initiation (sphere number) and the growth (sphere size) of spheres were reduced in Capan-1M9 cells upon rapamycin treatment, whereas the other cell lines only displayed reduced growth. Further analysis of secondary spheres showed that rapamycin treatment during primary sphere formation reduced the number of stem-like cells that could initiate the formation of secondary spheres, demonstrating the inhibition of self-renewal in the primary spheres. It was observed that the mTOR pathway functions to maintain the properties of stem-like pancreatic cancer cells.

The analysis of phosphorylation status revealed that the phosphorylation of mTOR effectors including 4E-BP1 (at sites T37/46), S6K (at site T389), and S6 ribosomal protein (at sites S240/244) was inhibited by rapamycin. These effectors function downstream of mTOR. On the contrary, the phosphorylation of Akt (at sites T308 and S473) was increased by rapamycin. Akt functions upstream of mTOR in the PI3K/Akt/mTOR pathway and may be activated by a feedback loop. This is the first report to demonstrate changes in the phosphorylation of mTOR effectors in pancreatic cancer stem-like cells by immunoblotting. These results suggest two possible mechanisms by which rapamycin treatment affects the properties of CSCs: the inhibition of downstream effectors and the activation of Akt (Supplementary Figure S8). Signaling to the pro-apoptotic factor BAD and to the FOXO transcription factors, which control cell viability and reactive oxygen species (ROS) levels, is known to be transduced directly from Akt and not through mTOR. In the cases of signaling directly from Akt, rapamycin causes the upregulation of signaling output from the PI3K/Akt pathway. However, aberrant activation of the PI3K/Akt pathway due to the activating mutation of PI3K and the amplification of Akt has been observed in a significant number of pancreatic cancer cases. In this regard, Akt activation is associated with malignant progression of the cancer, showing a gap between Akt activation and the suppression of CSC properties by rapamycin treatment. However, downstream signaling through mTOR was blocked by rapamycin even after the increase in Akt activity (Figure 6a).

In a recent report, mice expressing activated KRAS and lacking one allele of Pten in the pancreas displayed invasive pancreatic tumors and PI3K/Akt-dependent activation of NF-κB transcription^50^. Inhibition of NF-κB activity reduced the tumorigenic activity of cancer cells derived from these mice upon implantation into nude mice. Because Pten functions upstream of mTOR and negatively regulates PI3K/Akt signaling as a haploinsufficient tumor suppressor^50^, the loss of Pten is expected to result in the activation of mTOR. It is also noteworthy that the IκB kinase (IKK) is reported to interact with the mTORC1 complex in prostate cancer cells, and this interaction stimulates IKK activity towards the phosphorylation of IκB^51^. Because IκB is the negative regulator of the NF-κB pathway, phosphorylation and subsequent degradation of IκB increase the transcriptional activity of NF-κB. Rapamycin was shown to block NF-κB activity in these prostate cancer cells. Taken together, these findings may indicate that PI3K/Akt-dependent activation of NF-κB is mediated through mTOR (mTORC1) and that rapamycin treatment affects the properties of CSCs via inhibition of NF-κB activity. Further analysis is required to address whether this mechanism exists in pancreatic CSCs and functions to maintain stem cell-like properties.

In our study, mTOR inhibitor rapamycin inhibited the growth of xenografts of Capan-1M9 cells *in vivo*. These results were consistent with the *in vitro* findings. However, the percentage of CD133^+^ cells in xenografts showed the tendency to increase after rapamycin treatment, which was not shown *in vitro.* This discrepancy may reflect that rapamycin have multiple biological functions *in vivo*, not only for the cancer cells but also for their microenvironments. mTOR pathway has been implicated to the angiogenesis, HIF-1 regulation, and hypoxia response. Some of those phenomena are indicated to have a role in supporting and maintaining cancer stem cells. In this relation, it is interesting that the growth suppression was evident with 1 mg/kg/day, but it was not enhanced with 5 mg/kg/day treatment, whereas the CD133^+^ cell content was increased more gradually to 5 mg/kg/day. These differences in dose dependency could suggest that different mechanisms are underlying these two effects of rapamycin. Biological functions of mTOR pathway *in vivo*, including its function in the regulation of cancer microenvironment^52^, are remaining to be investigated.

The Hedgehog pathway has been implicated in the self-renewal and maintenance of pancreatic CSCs^53^,^54^,^55^. However, rapamycin treatment had different effects on Capan-1M9 cells than cyclopamine treatment, which inhibits Hedgehog signaling (Fig. 1). This finding indicates that the mTOR pathway has specific function(s) in these cells in addition to the shared function of the stemness pathways.

In a mouse leukemia model with deletion of Pten, which acts upstream of mTOR and attenuates the PI3K/Akt/mTOR signal, rapamycin not only depleted leukemia-initiating cells but also restored normal hematopoietic stem cell function^56^. Because normal hematopoietic stem cells were depleted in this model without rapamycin treatment, this finding indicates that mTOR has opposing effects on normal and malignant stem cells. Recent studies have also indicated that cancer stem cells of solid tumors display preferential sensitivity to the inhibition of the PI3K/Akt/mTOR pathway when compared with healthy stem cells^57^,^58^. CSCs are generally believed to share the regulatory molecular networks with normal stem cells. Therefore, the observations of the opposing effects of rapamycin on normal hematopoietic and leukemia stem cells or the preferential sensitivity of CSCs to mTOR inhibition compared with normal cells cannot be explained only by the shared function of the stemness pathways. In contrast, mTOR-specific function(s) could be a cause of the selective effects of rapamycin on normal and malignant stem cells. Recently, mTOR-mediated tumor suppressor responses were demonstrated in hematopoietic cells after Pten deletion, and the induction of tumor suppressors including p16^INK4a^, p53 and p19^Arf^ was implicated in leukemogenesis and the depletion of hematopoietic stem cells^59^. This could be an example of mTOR-specific function(s) in stem cells.

Therefore, the stem cell properties appear to be dependent not only on the shared function of the stemness pathways but also on the mTOR-specific function(s). If this hypothesis is correct, it will have significant therapeutic implications. In this regard, it is interesting that rapamycin and cyclopamine showed different effects on Capan-1M9 cells even though both inhibitors reduced the viability of CD133^+^ cells (Figure 1b). This result supports the implication of mTOR-specific function(s) in maintaining CSCs, which is a possible explanation for the effective elimination of pancreatic CSCs by the combination treatment targeting multiple pathways in the study by Mueller et al.^29^.

**Figure 8:**
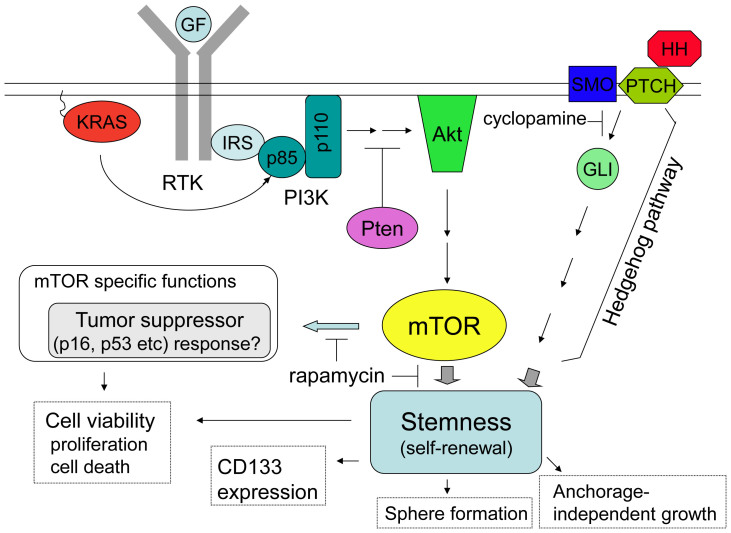
Proposed model for mTOR function in pancreatic cancer stem cells. The key genetic alteration driving the initiation of pancreatic ductal adenocarcinoma is activating KRAS mutations, which are found in > 90% of cases. mTOR functions downstream of KRAS to maintain the stemness of pancreatic CSCs. Therefore, the inhibition of mTOR by rapamycin reduced sphere formation and anchorage-independent cell growth and viability in CD133^+^ cells, which were likely CSCs. The Hedgehog pathway also functions to maintain pancreatic CSCs. However, the inhibition of these two pathways led to different cellular effects, suggesting that mTOR has specific function(s) in addition to the common function of maintaining the stemness of pancreatic CSCs. mTOR-mediated tumor suppressor responses have been reported in normal and cancer stem cells in another cell type^59^, showing a candidate of mTOR specific function(s) in pancreatic cancer cells.

The findings of the present study are summarized in Figure 8. The mTOR pathway was shown to maintain the stem cell-like properties of pancreatic cancer cells. Sphere formation under stem cell culture conditions and anchorage-independent colony formation were both dependent on this pathway. Inhibition of the pathway by rapamycin effectively reduced the viability of cancer stem-like cells. The rapamycin treatment showed different cellular effects compared with the inhibition of other stemness pathways, indicating the existence of mTOR-specific function(s) in addition to the common function(s) of the stemness pathways in the maintenance of stem cell-like properties. In conclusion, targeting the mTOR pathway might be an effective therapeutic approach to eliminate pancreatic cancer stem cells.

## Methods

### Cell culture and reagents

The human pancreatic cancer cell line PANC-1 was obtained from the American Type Culture Collection (ATCC, Manassas, VA, USA). Capan-1M9 cells were established as described previously^27^. PCK-2 cells were established from malignant ascites of a pancreatic cancer patient in our laboratory. We used the second-passage PCK-2 cells for this study. The cells were cultured in DMEM/F12 medium (Sigma, St. Louis, MO, USA) containing 10% FBS (HANA-NESCO, Tokyo, Japan) supplemented with 100 units/ml penicillin and 100 mg/ml streptomycin at 37°C in 5% CO_2_. Rapamycin was purchased from Sigma (St. Louis, MO, USA). For inhibitor treatment, Capan-1M9 cells were plated at a density of 2 × 10^5^ cells per 100-mm dish and incubated for 48 hr at 37°C. The medium was replaced with medium containing rapamycin or other reagents, and the dish was then incubated at 37°C for another 72 hr.

### Flow cytometric analysis

A total of 10^6^ cells were suspended in 100 μl PBS containing 0.5% BSA. The mouse anti-human CD133 mAb (either R-phycoerythrin (PE)-conjugated or allophycocyanin (APC)-conjugated, Miltenyi Biotec, Cologne, Germany) was appropriately diluted in the FcR blocking reagent to a final volume of 20 μl. The antibody was added to the cells, and the mixture was incubated on ice for 10–20 min in the dark. The cells were washed and resuspended in a suitable amount of buffer for analysis by flow cytometry. Flow cytometric analyses were carried out with a FACSAria flow cytometer (Becton Dickinson, Franklin Lake, NJ, USA). Dead cells were excluded by 7-amino-actinomycin-D (BD Pharmingen, San Diego, CA, USA) staining.

### MTT cell viability assay

Single cells were resuspended in fresh medium at a concentration of 2 × 10^3^ cells/100 μl and seeded in a 96-well plate. The cells were incubated for 48 hr at 37°C, and the medium was then replaced with fresh medium containing rapamycin or other reagents. The plate was then incubated at 37°C for another 72 hr. For the MTT assay, 15 μl of 3-(4,5-dimethylthiazol-2-yl)-2,5-diphenyltetrazolium (MTT; 1 mg/ml in PBS) was added to each well, and the plate was incubated for an additional three hr at 37°C. The medium was removed, and 100 μl of DMSO was added. The absorbance was measured at 570 nm using a microplate reader.

### Sphere formation assay

Spheres were cultured in DMEM/F12 serum-free medium supplemented with epidermal growth factor (20 ng/ml; Cell Signaling Tech, Denver, MA, USA), basic fibroblast growth factor (10 ng/ml; PeproTech, London, UK), and B27 supplement (1:50; Gibco, Grand Island, NY, USA). The cells were cultured in 96-well ultra-low attachment plates for eight days.

### Anchorage- independent colony formation assay

Each well (16 mm) of 24-well plates was coated with 0.5 ml bottom agar-medium mixture (DMEM/F12, 10% FCS, 0.5% agar). After the bottom layer was solidified, 0.5 ml of top agar-medium mixture (DMEM/F12, 10% FCS, 0.33% agar) containing 10^3^ cells was added, and the dishes were incubated at 37°C for two to four weeks. The numbers of colonies were counted under a microscope.

### Immunoblotting

After drug treatment, the cells were washed with ice-cold PBS, mixed with SDS sample buffer^60^, lysed, and boiled for five min. After SDS-PAGE (5–20% polyacrylamide), the proteins were transferred to nitrocellulose membranes. The membranes were blocked with 5% nonfat milk in PBS and then incubated with primary antibodies. The membranes were then incubated with peroxidase-conjugated secondary antibodies (Jackson ImmunoResearch Laboratories, West Grove, PA, USA). After washing with buffer containing 0.1% Tween 20, the protein bands were visualized with an ECL detection kit (Amersham, Little Chalfont, UK). The proteins were detected with the following antibodies: anti-phospho-4E-BP1 (T37/46), anti-4E-BP1, anti-phospho-p70/p85 S6 Kinase (S6K, T389), anti-p70/p85 S6 Kinase (S6K), anti-phospho-S6 ribosomal protein (S240/244), anti-S6 ribosomal protein, anti-phospho-Akt (T308), anti-phospho-Akt (S473), anti-Akt (all from Cell Signaling Technology, Beverly, MA, USA), anti-phospho-ERK1/2, and anti-ERK1/2 (Santa Cruz Biotechnology, Santa Cruz, CA, USA).

### *In vivo* treatment of xenograft with rapamycin

The animal study was approved by the Committee on the Use of Live Animals for Teaching and Research of Kagoshima University. BALB/c nu/nu (nude) mice were purchased from CLEA Japan (Tokyo, Japan). For *in vivo* treatment of rapamycin, nude mice were randomly assigned to three groups at two weeks after s.c. injection of Capan-1M9 cells (10^6^). Mice received treatments of vehicle (5% PEG400, 5% Tween-80 and 4% ethanol, i.p.), 1 mg/kg/day rapamycin (i.p.), or 5 mg/kg/day rapamycin (i.p.) for 17 days. The tumors were measured after a 4-day interval; the tumor volume was calculated as follows: tumor volume = length × width^2^/2.

### Statistical analyses

The results are expressed as the mean ± s.d. Comparisons between the averages of two groups were evaluated using a two-tailed Student's *t*-test. *P* < 0.05 was considered statistically significant.

## Author Contributions

S.T. and S.M. designed research; S.M. and S.T. wrote the main manuscript text; Q.D., Y.M., T.K. and S.M. prepared figure 1; Q.D., K.T. and S.M. prepared figure 3; T.K. and S.M. prepared figure 6; S.T. prepared figure S2; K.T. prepared figures S1; S.T., K.T. and S.M. prepared figure 7 and figure S7. All other figures were prepared by S.M.; and all authors reviewed the manuscript.

## Supplementary Material

Supplementary Information Supplementary Information
