# Supplementary material for: mTOR plays critical roles in pancreatic cancer stem cells through specific and stemness-related functions
Source: Sci Rep. 2013 Nov 15;3:3230. doi: 10.1038/srep03230 (PMC3828572; doi:10.1038/srep03230)
Supplement: Supplementary Information [file srep03230-s1.pdf]

Supplementary Information

mTOR plays critical roles in pancreatic cancer stem cells through specific and stemness-related functions

Shyuichiro Matsubara[1], *Qiang Ding*[1], *Yumi Miyazaki*[1], *Taisaku Kuwahata*[2], *Koichiro Tsukasa*[1] *and Sonshin Takao*[1]

[1]Cancer and Regenerative Medicine, Frontier Biomedical Science and Swine Research Center, [2]Department of Digestive Surgery, Kagoshima University Graduate School of Medical and Dental Sciences 8-35-1, Sakuragaoka, Kagoshima, 890-8520, Japan

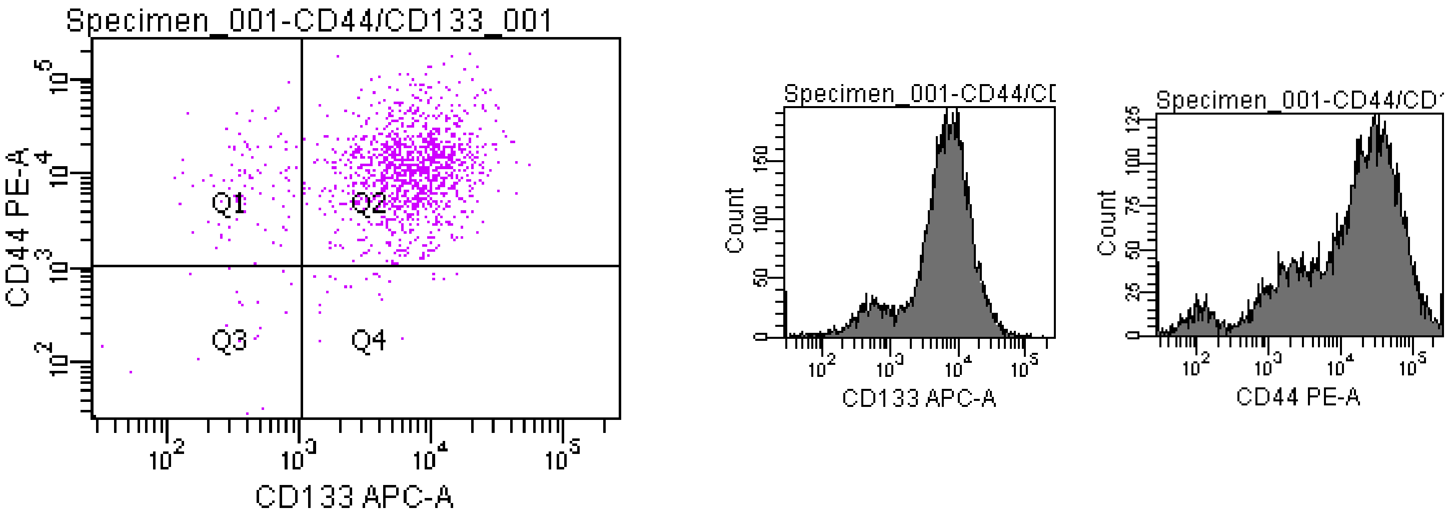

Supplementary Figure S1. FACS analysis of Capan-1M9 cells (untreated cells).

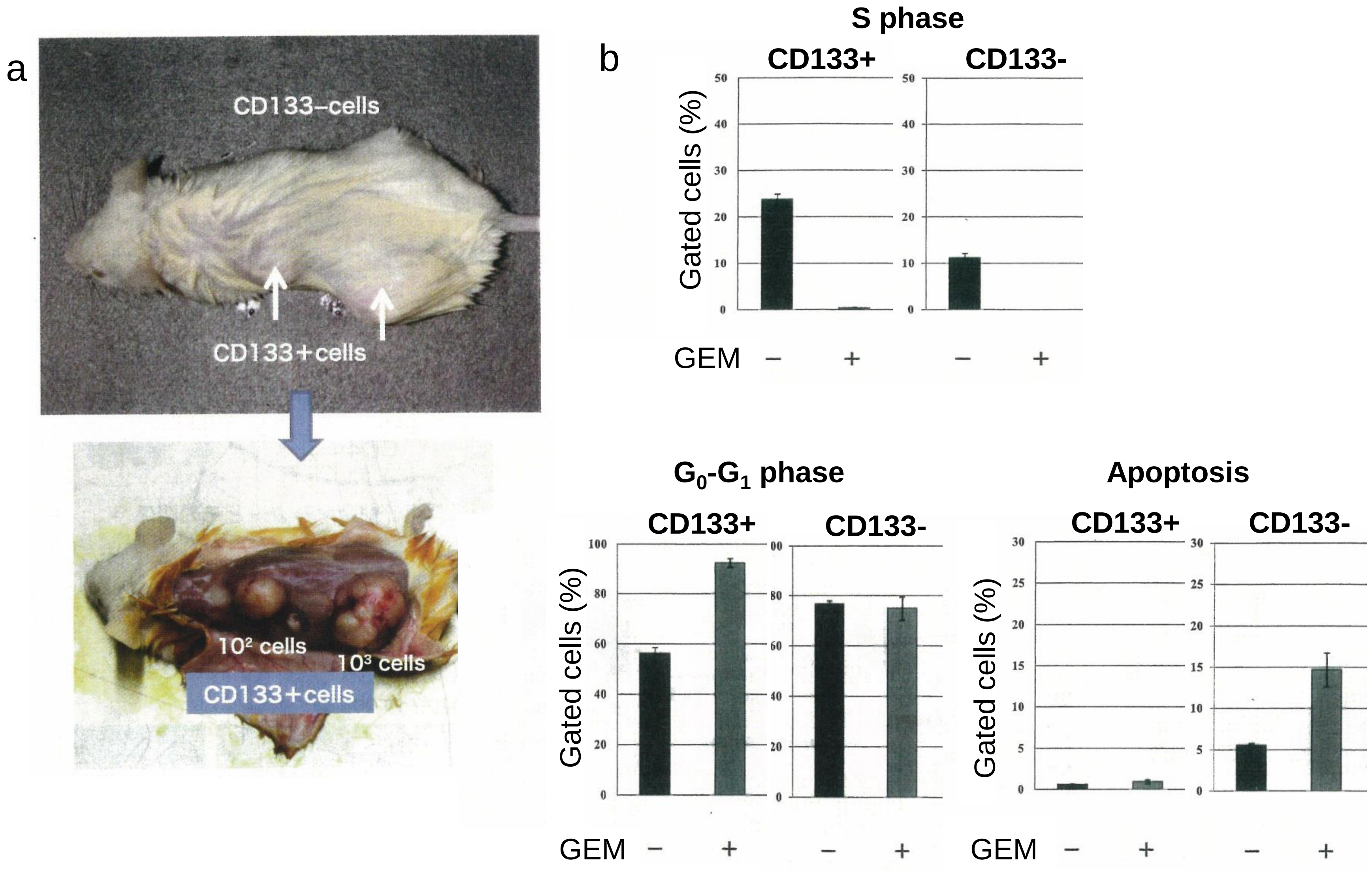

Supplementary Figure S2. $CD133^+$ cells from the pancreatic cancer cell line Capan-1 show cancer stem cell-like properties. (a) The tumorigenicity of $CD133^+$ and $CD133^-$ cells in NOD/SCID mice. (b) Comparison of the distribution of $CD133^+$ and $CD133^-$ cells in the G0/G1 phase and the S phase. Apoptosis was also analyzed by the BrdU assay after gemcitabine (GEM) treatment (100 ng/ml). (c) Gemcitabine treatment (100 ng/ml) increases the percentage of $CD133^+$ cells in Capan-1.

Supplementary figure S2

c

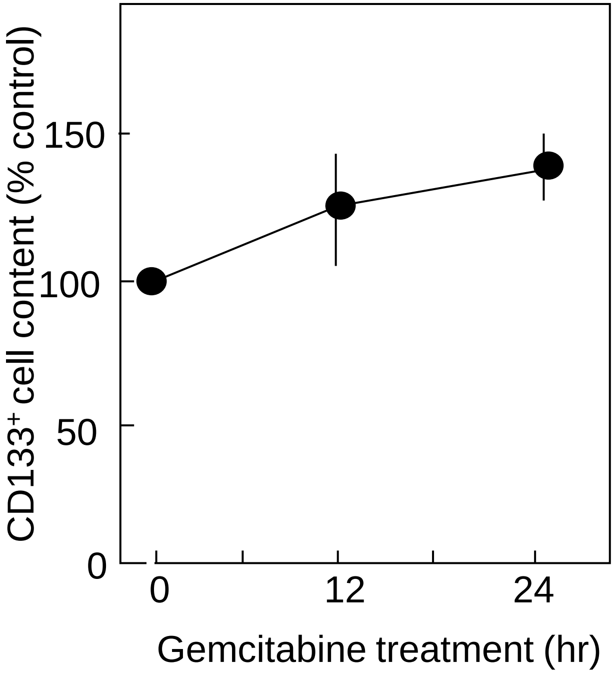

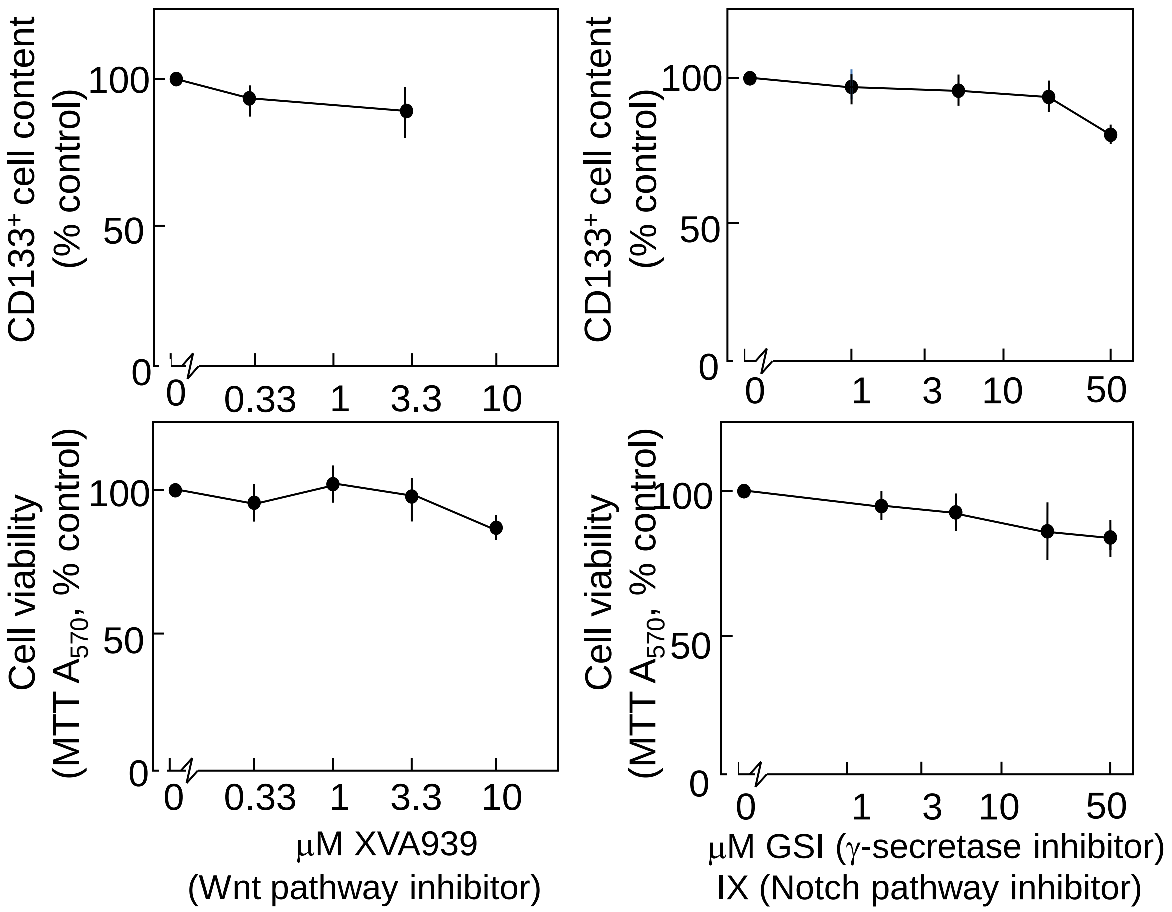

Supplementary Figure S3. Inhibition of Wnt or Notch did not significantly affect either the percentage of CD133^{+} cells or the viability of Capan-1M9 cells. The effects of the Wnt pathway inhibitor XVA939 and the Notch inhibitor GSI IX on CD133^{+} cell percentage and cell viability were determined and presented as Figure 1 (a).

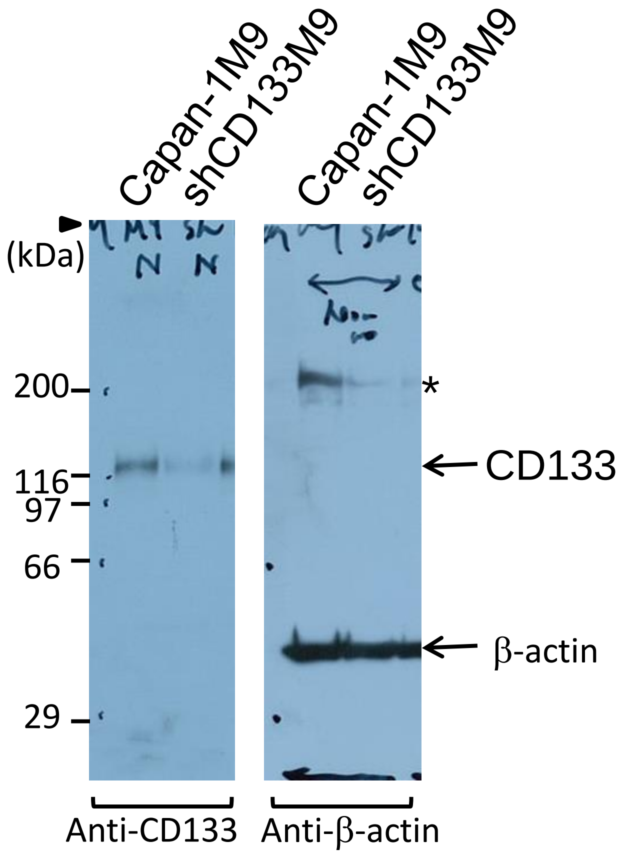

Supplementary Figure S4. Full size blots of the immunoblot detection shown in Figure 2a.

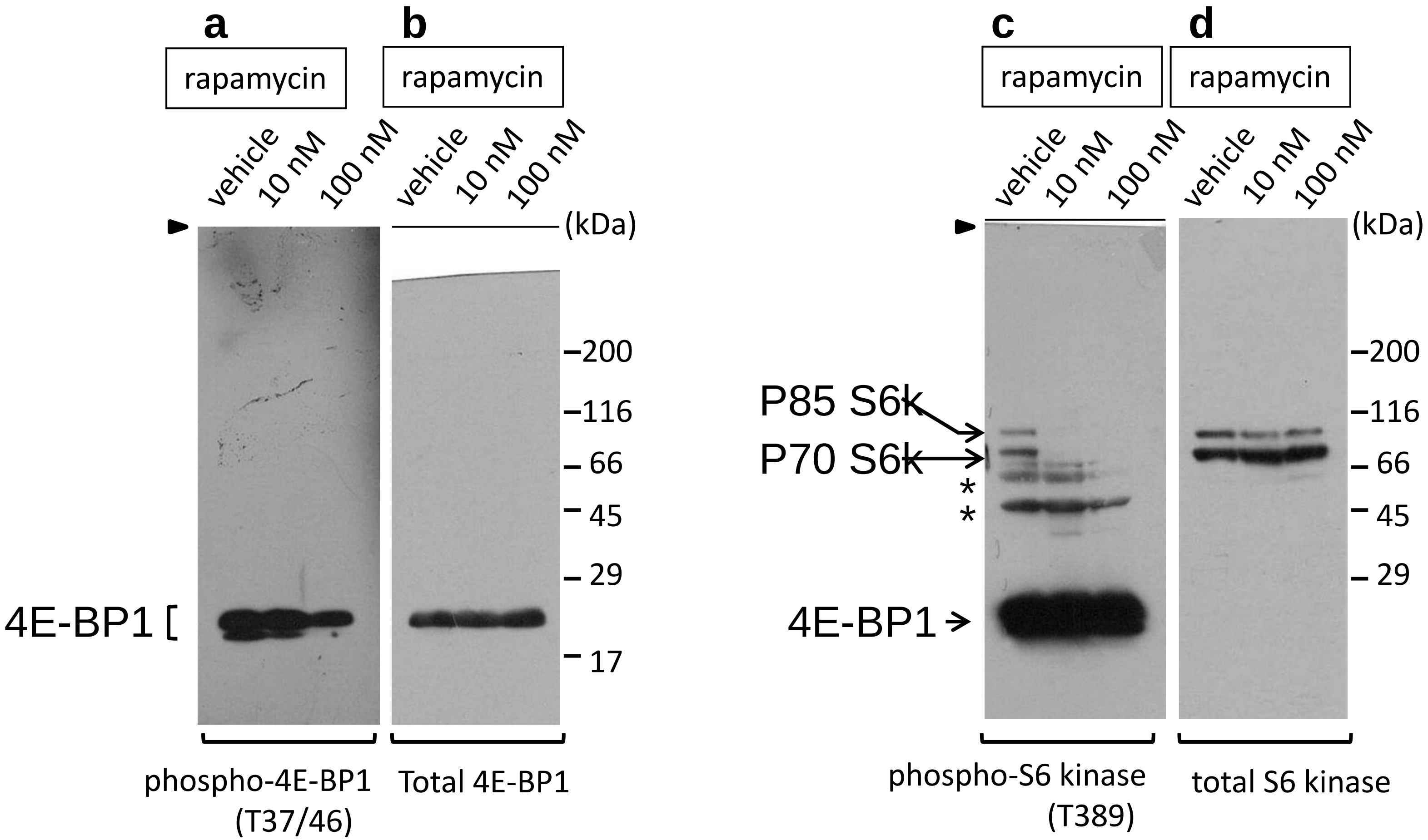

Supplementary Figure S5. Full size blots of the immunoblot detection shown in Figure 6. * indicates non-specific bands. The same filters were used several times with different antibodies, therefore the signal in the previous detection was appeared in (c), (d), (e), (f) and (k).

Supplementary figure S5 (continued)

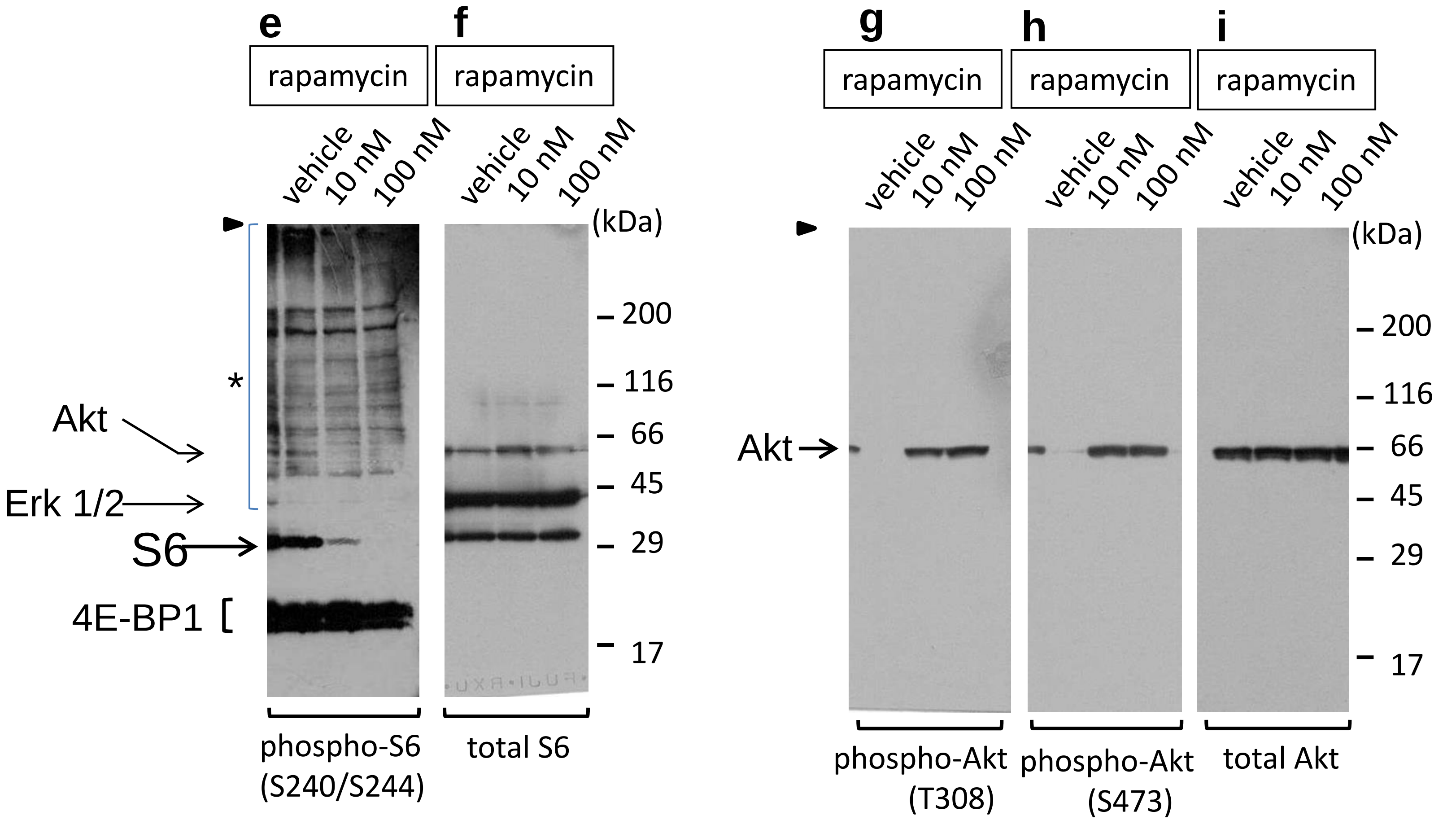

Supplementary figure S5 (continued)

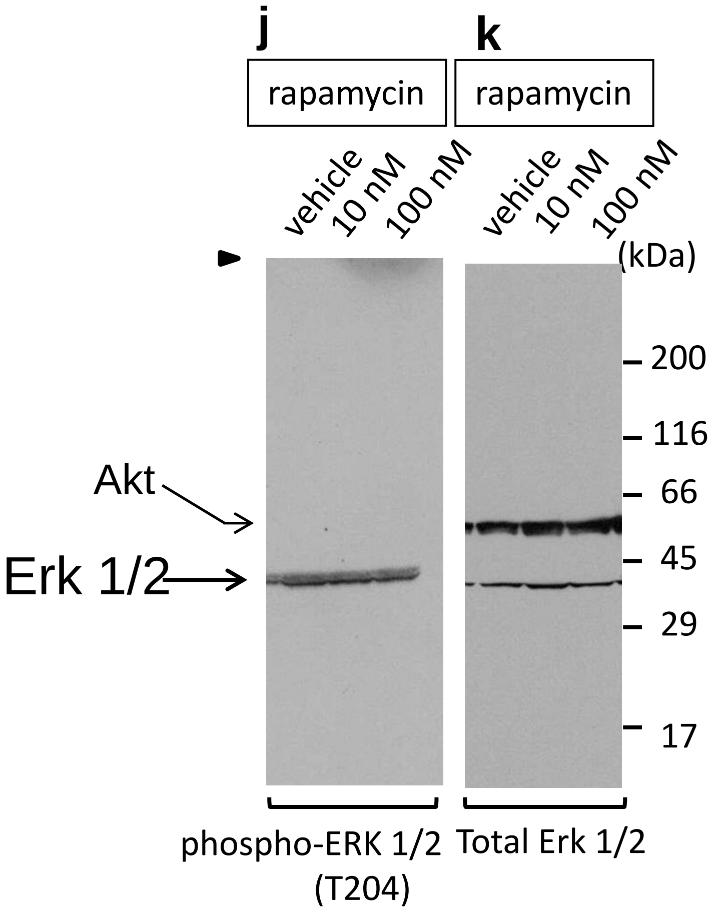

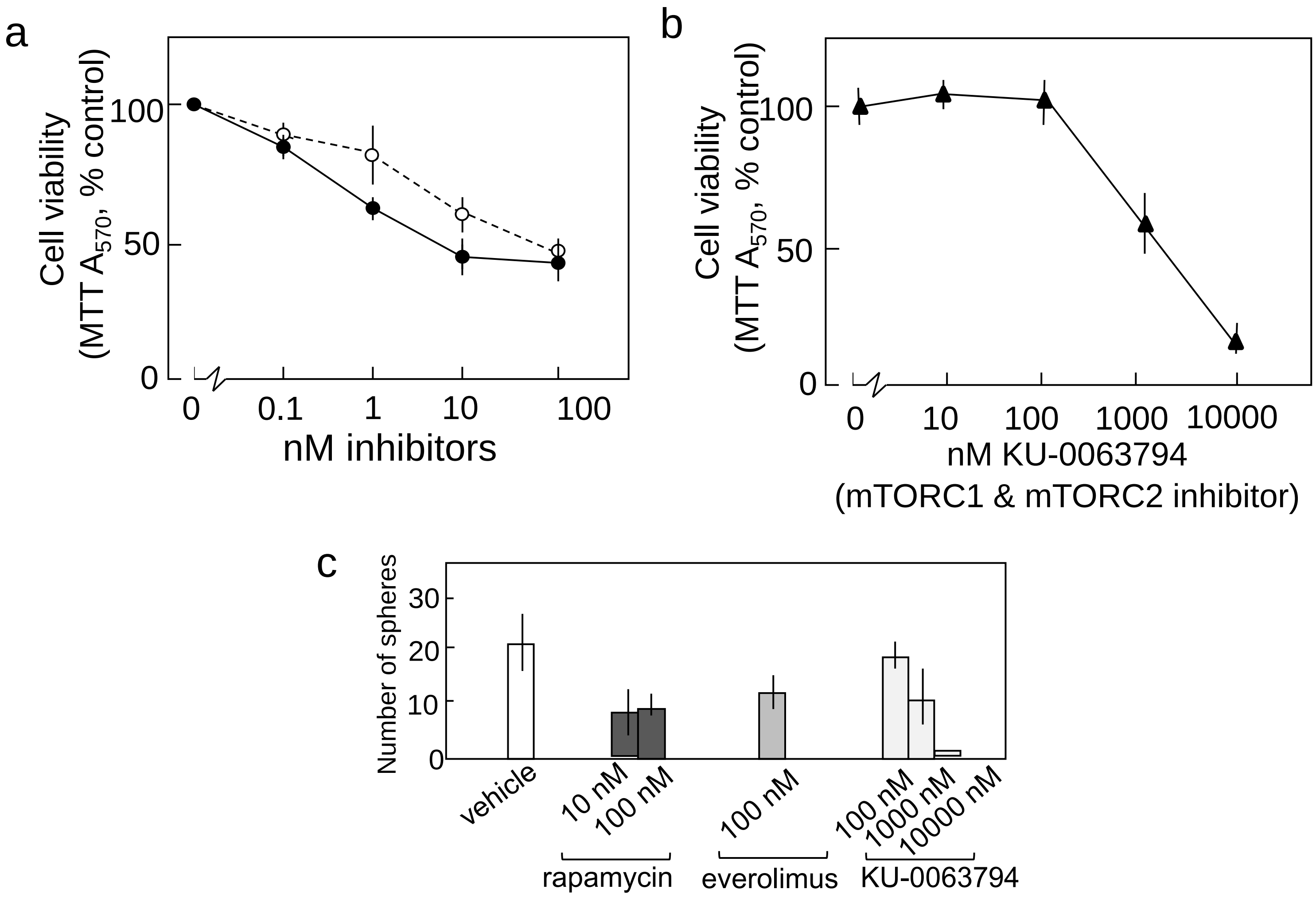

Supplementary Figure S6. mTORC1/mTORC2 dual inhibitor KU-0063794 reduces the cell viability and sphere formation of Capan-1M9 cells but the inhibition kinetics is different from rapamycin. Rapamycin and its derivatives directly inhibit mTORC1 and their effects on mTORC2 assembly are reported in some cell lines. (a) Effects of rapamycin (closed circle) and everolimus (open circle) and (b) of KU-0063794 (closed triangle) on cell viability were determined and presented as Figure 1 (a). (c) Effects of rapamycin, everolimus and KU-0063794 on sphere formation were determined and presented as Figure 2 (e).

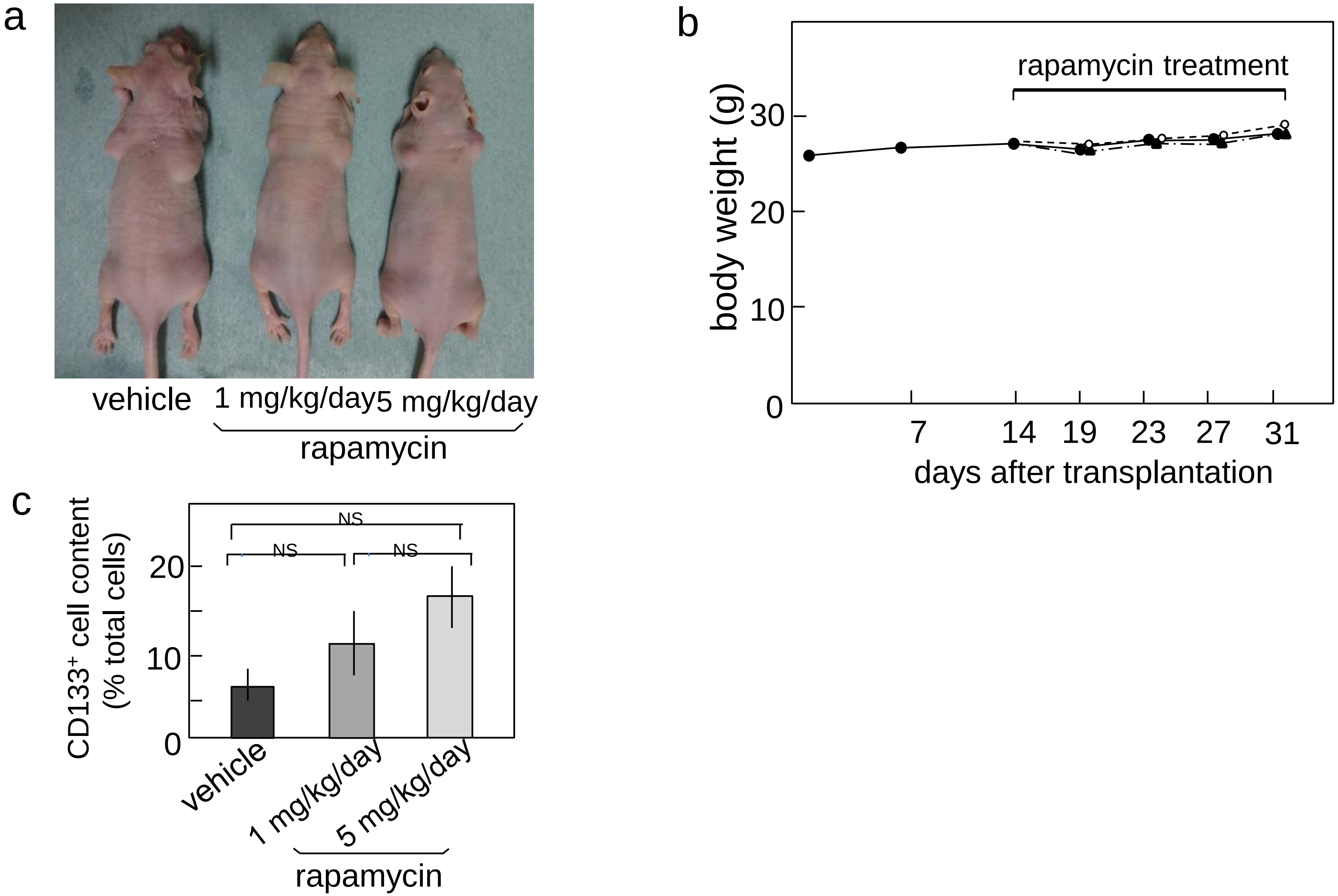

Supplementary Figure S7. Effect of rapamycin on the xenografted Capan-1M9 tumor in nude mice.
(a) Xenograft tumors with rapamycin treatment were smaller than those with controls in nude mice. (b) Body weight of nude mice were not significantly different among the three treatments, vehicle (open circle), 1 mg/kg/day (closed circle), or 5 mg/kg/day (closed triangle) rapamycin. (c) The flow cytometric analysis of xenograft cells after enzyme dissociation. NS $P > 0.05$.

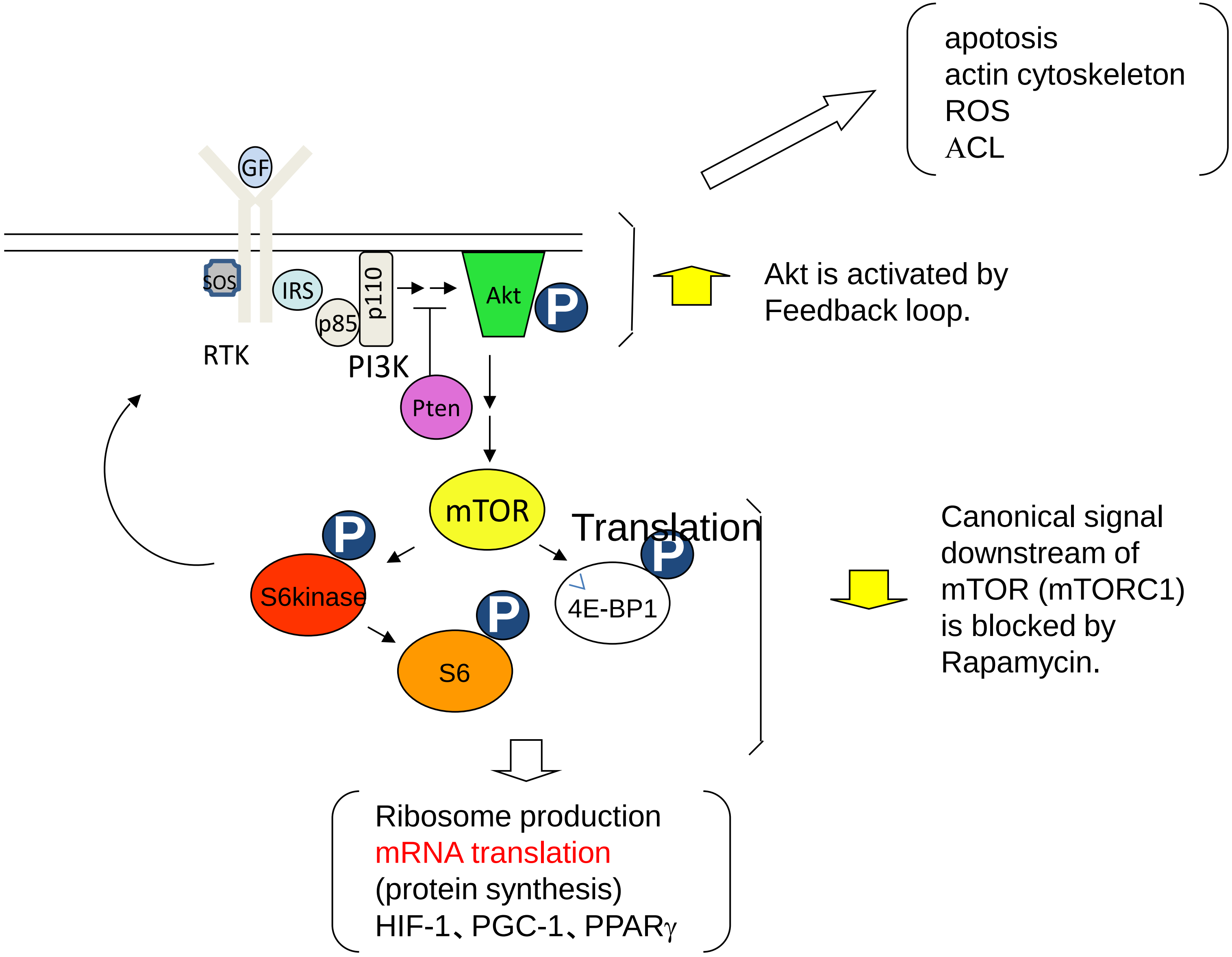

Supplementary Figure S8. Signaling output from the PI3K/Akt/mTOR pathway after rapamycin treatment.
